# Recrystallization Texture Evolution in Fe–3.0 wt.% Si Hot-Rolled Silicon Steel Sheet by Quasi In Situ EBSD Analysis

**DOI:** 10.3390/ma19040650

**Published:** 2026-02-08

**Authors:** Fang Zhang, Huabing Zhang, Songtao Chang, Gengsheng Cao, Yuhui Sha, Liang Zuo

**Affiliations:** 1 Key Laboratory for Anisotropy and Texture of Materials, Ministry of Education, Northeastern University, Shenyang 110819, China; zhanghuabing@baosteel.com (H.Z.); cst_chang@163.com (S.C.); cgsformal94@163.com (G.C.); lzuo@mail.neu.edu.cn (L.Z.); 2 Central Research Institute, Baoshan Iron & Steel Cooperation Limited, Shanghai 201900, China

**Keywords:** Fe–3.0%Si, hot-rolled steel sheet, quasi in situ EBSD analysis, recrystallization, Goss texture, nucleation mechanism

## Abstract

The microstructural and textural evolution in hot-rolled Fe–3.0 wt.% Si steel sheets was investigated by quasi in situ electron backscatter diffraction (EBSD) analysis. During recrystallization, the Goss texture intensity in the surface region remains essentially unchanged, whereas the α and α* textures are strengthened. In the center region, the α texture weakens, and the α* texture shows little variation, while the Goss texture becomes intensified. In the surface region, {112}<110> recrystallized grains nucleate by consuming deformed matrices with orientations near {114}<221> and {110}<112>. Recrystallized {114}<481> and {001}<210> grains consume deformed matrices near {114}<221> and Goss orientations, while Goss grains nucleate by consuming Goss-oriented deformed matrices. In the center region, {112}<110>, {114}<481>, and {001}<210> recrystallized grains nucleate and grow by consuming α and λ type deformed matrices, whereas Goss recrystallized grains preferentially consume deformed matrices with orientations of {111}<112>.

## 1. Introduction

Silicon steel is an indispensable soft-magnetic material widely used in the electrical power, electronics, and new-energy industries. Hot-rolled silicon steel sheets need typically undergo recrystallization annealing to prepare suitable microstructure and texture conditions for subsequent cold rolling [1,2,3,4]. In industrial production, this annealing treatment to hot-rolled sheets is termed normalization, which alters the texture components and their intensities, and subsequently affects the microstructure and texture development during cold deformation and final annealing through inheritance effects [5,6,7]. From the perspective of microstructure and texture evolution throughout the entire processing route of silicon steel, normalization after hot rolling plays a crucial role in subsequent texture control. During normalization, the selection of recrystallization nucleation sites, texture inheritance, and orientation-dependent preferential growth directly determine the orientations and their spatial distribution across the thickness of hot rolled sheets [8,9,10]. These features, in turn, profoundly influence the orientation rotation and the distribution of stored energy during cold rolling, as well as the texture evolution during subsequent recrystallization and secondary recrystallization [11,12,13]. In particular, the key orientations formed or strengthened during normalization such as Goss and α* (φ_1_ ≈ 20°, Φ = 0~55°, φ_2_ = 45°) often dominate the final texture of finished silicon steel sheets [14,15]. Conversely, if unfavorable orientations are not effectively suppressed at this stage, their detrimental effects tend to accumulate throughout the subsequent processing steps. Therefore, normalization of hot-rolled sheets essentially serves as a texture pre-design step and constitutes a critical prerequisite for achieving controllable texture evolution and optimized magnetic properties in silicon steel.

Lin et al. [16] reported that during normalization, the Goss texture in the surface region becomes strengthened, while the α texture gradually transforms into the α* texture. Mehdi et al. [17] showed that the surface texture of 3.2 wt.% Si steel hot bands mainly consists of Copper ({112}<111>), Brass ({110}<112>), and Goss components ({110}<001>), whereas the center region is characterized by strong λ and weak γ textures. After annealing at 860 °C, the λ texture nearly disappears, and a {114}<261> component close to the α* texture forms, while the Copper, Brass, and Goss components are markedly weakened.

Fukagawa et al. [18] observed numerous Goss grains at 1/10 thickness of normalized grain-oriented silicon steel, while strong α and γ textures are present at 1/2 thickness. Park et al. [19] reported that a weak Goss texture forms in the surface region, whereas strong α and weak γ textures develop in the inner region. Shin et al. [20] found that grain growth during normalization has limited influence on texture inhomogeneity. Tang et al. [21] explained the dominance of the {114}<481> component in the center region after normalization using a selective-growth mechanism.

Wu et al. [22] demonstrated that normalization markedly modifies the hot-rolled texture. After normalization at 940 °C, the primary textures are Goss and rotated cube; after annealing at 980 °C, the texture becomes further weakened, dominated by weak Goss and {114}<481> components. Hu et al. [23] reported that the hot-rolled texture consists mainly of γ, λ, and α components, with γ and α being dominant. After normalization, the texture distribution becomes more random. At 850 °C, the Goss texture is the dominant component, but with increasing annealing temperature, the Goss intensity decreases while the γ texture becomes strengthened. After normalization at 1000 °C, the Cube component becomes enhanced and the Goss nearly disappears; at 1050 °C, γ becomes the strongest texture component.

During normalization annealing of hot-rolled silicon steel, the recrystallization texture evolution is governed by the competition between two classic mechanisms: oriented nucleation (ON) and oriented growth (OG) [24,25]. The ON theory posits that specific orientations nucleate more frequently within the deformed matrix and determine the recrystallization texture by a higher number of grains [26]. In contrast, the OG theory suggests that grains with specific grain boundary misorientation grow faster into the deformed matrix, thus dominating the recrystallization texture by size advantage [27,28]. For the diversity of texture evolution during the annealing process of hot-rolled sheets, effective control of the recrystallization texture requires a thorough understanding of its origin and evolution. For example, a Goss texture in the subsurface of normalized sheets plays a critical role for the development of a sharp secondary recrystallization Goss texture in grain-oriented silicon steel. Although the classic texture formation theories are well-established, systematic investigations that resolve the nucleation and texture evolution of individual texture components across different through-thickness layers during normalization remain limited.

Furthermore, most previous studies relied on conventional ex situ EBSD techniques, which can provide statistical texture information but fail to directly link specific recrystallized grains to their parent deformed matrices. Consequently, the local orientation relationship between the nucleus and the consumed matrix, which is critical for distinguishing between nucleation-controlled and growth-controlled mechanisms, is often lost. In situ EBSD analysis can overcome this limitation by tracking the same region throughout the annealing process, enabling the direct observation of nucleation sites and the explicit identification of the deformed matrix consumed by growing grains.

In this work, a Fe–3.0 wt.% Si hot-rolled sheet is employed, which maintains a single-phase ferritic structure during annealing, and exhibits a severe texture gradient from the surface to the center in hot-rolled sheets. An in situ EBSD technique is used to identify the nucleation sites within specific deformed regions and the deformed orientations preferentially consumed by growing grains. The results can clarify the origin and formation mechanisms of the major recrystallization texture components developed during the normalization of hot-rolled sheets.

## 2. Materials and Methods

A Fe–3.0 wt.% Si hot-rolled steel sheet with a thickness of 2.3 mm was used as the starting material. The chemical composition of the steel is listed in Table 1.

After mechanical polishing, the ND–RD plane of the hot-rolled samples was electrolytically polished in a solution of 92 vol.% ethanol and 8 vol.% perchloric acid. EBSD characterization was carried out using a Crossbeam−550 scanning electron microscope. The samples were annealed in a vacuum furnace at 850 °C for 30 s, 30 s, 35 s, 40 s, 60 s, 90 s, and 180 s, respectively. After each annealing step, EBSD measurements were conducted on the same area to enable quasi in situ observation. The characterization was performed at an accelerating voltage of 20 kV, a working distance of 165 mm, and a step size of 1.5 μm. The quasi in situ EBSD data were analyzed using MTEX 5.10.0 processing.

Recrystallized grains were identified using a multi-parameter criterion to ensure clear separation from the deformed matrix. Specifically, grains were defined as recrystallized if they possessed an average intragranular misorientation below 1° and a Grain Orientation Spread (GOS) of less than 3°. To further refine the identification, a Kernel Average Misorientation (KAM) threshold of <0.1° was applied to confirm low local dislocation density. Additionally, to exclude noise and artifacts, recrystallized grains were required to have a minimum size of 9 pixels and an aspect ratio smaller than 3 and be completely surrounded by grain boundaries with misorientation angles larger than 5°.

**Figure 1:**
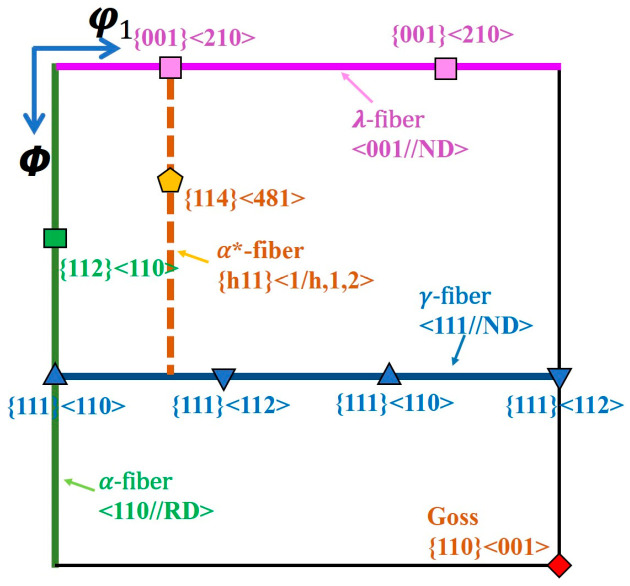
The standard positions of some orientations on φ_2_ = 45° section. The α*-fiber is defined as the orientation line deviating by 18° from the α-fiber.

Figure 1 illustrates a standard ODF reference map for verifying the orientations of main texture components and analyzing the orientation relationships during recrystallization in this study. These components were defined using a maximum deviation angle of 15°.

## 3. Results

### 3.1. Microstructure and Texture of the Hot-Rolled Sheet

**Figure 2:**
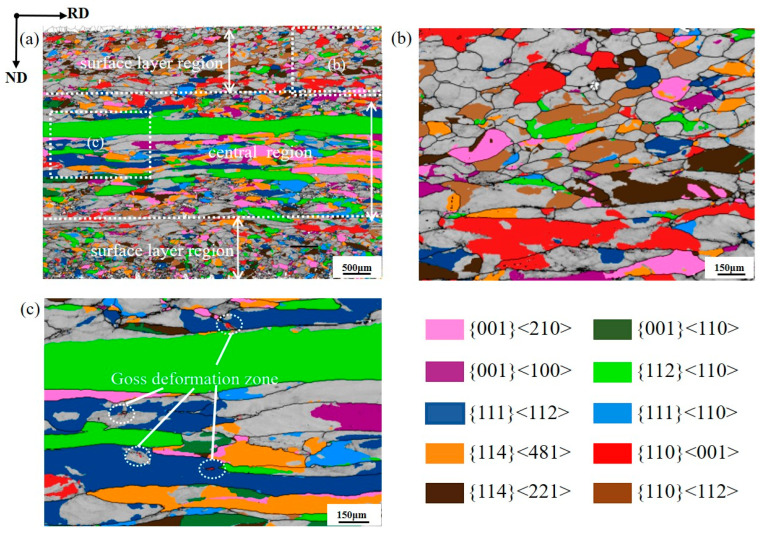
Orientation imaging map (OIM) of the major texture components in the hot-rolled silicon steel sheet (**a**) and the corresponding enlarged views (**b**,**c**).

Figure 2 presents the orientation imaging maps of the major texture components in the hot-rolled silicon steel sheet. A pronounced microstructural gradient is observed across the sheet thickness. In the surface region, the deformation temperature is relatively low and the material is subjected to intense shear imposed by the hot-rolling rolls. As a result, both dynamic and static recrystallization occur during hot rolling, leading to the formation of fine deformed grains; the Goss-deformed areas occupy a relatively large fraction and typically appear in the form of Goss-deformed grains. In contrast, the center region experiences a higher deformation temperature and is mainly deformed under plane-strain conditions, resulting in elongated deformed grains. Goss-deformed grains are rarely observed in the center region; instead, small deformed areas of Goss are distributed along grain boundaries and within the interiors of {111}<112> deformed grains (Figure 2c).

**Figure 3:**
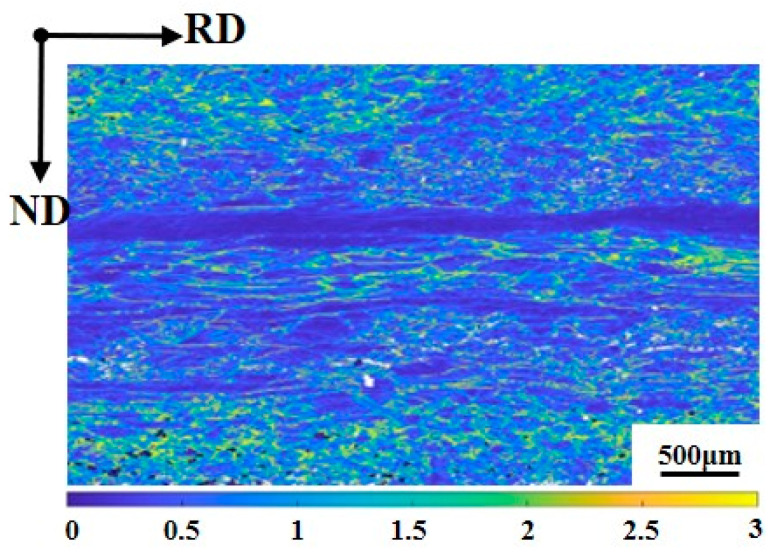
Kernel average misorientation (KAM) distribution map in the hot-rolled silicon steel sheet.

To address the stored energy distribution before annealing, KAM maps for the surface and center regions were obtained, shown in Figure 3. The surface region exhibits significantly higher average KAM values compared to the center, indicating a greater density of geometrically necessary dislocations and higher stored energy induced by intense shear strain during hot rolling. This high and relatively uniform stored energy in the surface promotes rapid, massive nucleation, whereas the lower and more localized stored energy in the center leads to a lower nucleation frequency and provides space for orientation-dependent growth.

**Figure 4:**
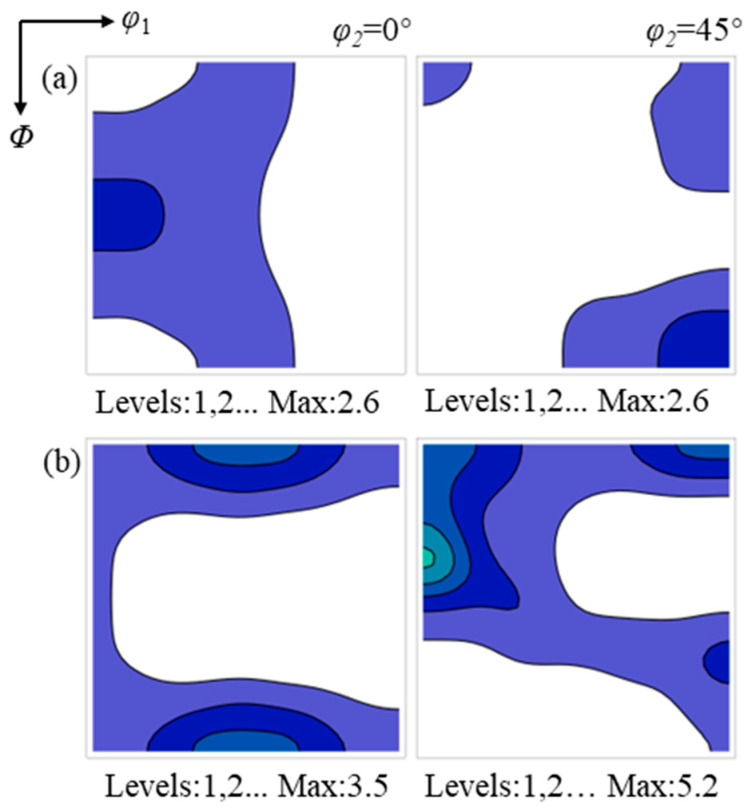
ODF sections of the surface region (**a**) and the center region (**b**) of the hot-rolled silicon steel sheet. The color intensity indicates the relative texture strength.

Figure 4 shows the ODF sections at φ_2_ = 0° and 45° for the surface and center regions of the hot-rolled sheet. The temperature and strain gradients during hot rolling lead to a pronounced macroscopic texture gradient in the hot-rolled sheet. The near-surface region is characterized by discontinuous ε (<110>//TD) and ζ (<110>//ND) shear textures, with maximum texture intensity at the Goss orientation. In contrast, the center region exhibits a strong α texture and a relatively weak γ texture. The intensity peak of the α texture is located at {112}<110>, whereas the γ texture is uniformly distributed along the corresponding orientation fiber. The Goss texture in the center region is significantly weaker than that in the surface region.

### 3.2. Microstructural Evolution of the Hot-Rolled Sheet During Annealing

During annealing, recrystallization proceeds much faster in the surface region than in the center region. At 95 s, the recrystallized area fraction in the surface region reaches ~75%, while that in the center region is only ~38%, indicating that recrystallization in the surface region is nucleation-controlled, whereas that in the center region is growth-controlled at early stages.

Texture evolution analysis shows that the Goss texture in the surface region remains nearly unchanged during annealing, while the α* texture is strengthened. In contrast, the α and γ textures weaken in the center region, while the Goss texture becomes progressively enhanced.

**Figure 5:**
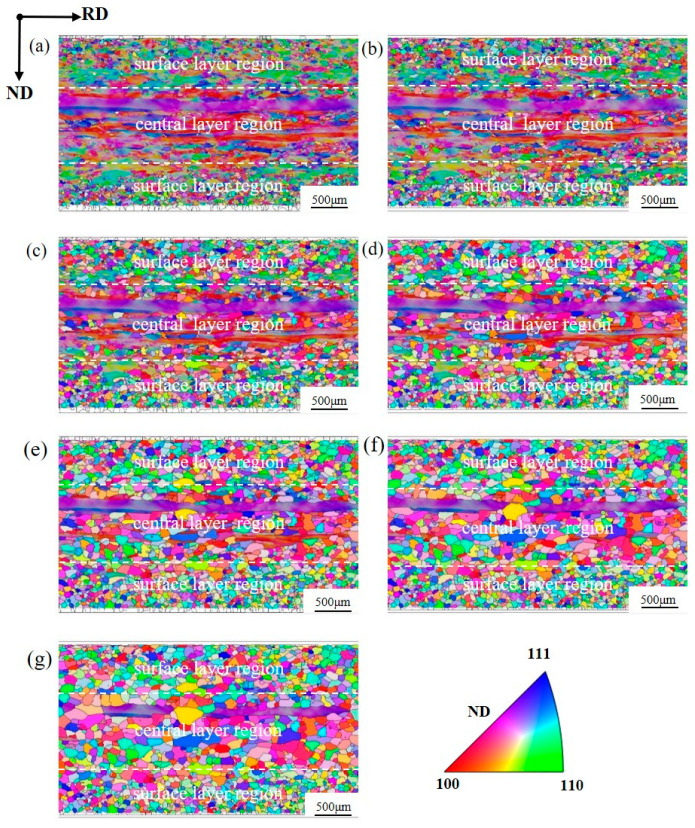
Microstructure evolution of hot-rolled sheet during recrystallization: (**a**) 30 s; (**b**) 60 s; (**c**) 95 s; (**d**) 135 s; (**e**) 195 s; (**f**) 285 s; and (**g**) 465 s.

The hot-rolled sheet was annealed at 850 °C for different times, and the microstructure and texture after each annealing were characterized by EBSD. Figure 5 illustrates the microstructural evolution of the hot-rolled sheet during annealing; it is obvious that the microstructural evolution during annealing differs markedly between the surface and center regions.

After annealing for 30 s, a considerable number of recrystallized grains form in the surface region, whereas the number of recrystallized grains in the center region is markedly lower. With increasing annealing time, the number of recrystallized grains increases in both regions, but the increase is much more pronounced in the surface region.

When the annealing time reaches 95 s, a high fraction of recrystallized grains in the surface region come into mutual contact, while most recrystallized grains in the center region remain surrounded by deformed grains. During subsequent annealing, the driving force for recrystallized grain growth in the surface region shifts from deformation-stored energy to grain boundary energy, resulting in a pronounced slowdown in the grain growth rate after 95 s. In contrast, due to the low nucleation density in the center region, recrystallized grains continue to grow under the driving force of deformation-stored energy.

After annealing for 465 s, recrystallization is completed in the surface region, whereas some elongated deformed grains still remain in the center region. The low intragranular misorientation and correspondingly low stored energy within these elongated deformed grains cause them to persist during the later stages of annealing without being consumed. Recrystallized grains in the surface region are small and relatively uniform in size, whereas those in the center region are larger and more scattered. In the center region, some recrystallized grains exhibit a pronounced size advantage, indicating that recrystallized grains in this region undergo extensive growth during annealing and that certain grains may possess a growth advantage.

During recrystallization, the recrystallized area fraction and the number of recrystallized grains in the surface region remain consistently much higher than those in the center region. At an annealing time of 95 s, the number of recrystallized grains in both regions reaches a maximum value, with the surface region exhibiting approximately 2.5 times more recrystallized grains than the center region.

With further annealing, grain growth driven by grain boundary energy occurs in both regions, leading to a slight decrease in the number of recrystallized grains. When the recrystallized grain number reaches its peak, the recrystallized area fraction is about 75% in the surface region, whereas it is only 38% in the center region. These results indicate that the recrystallization process in the surface region is dominated by grain nucleation, whereas that in the center region is primarily governed by grain growth.

### 3.3. Texture Evolution During Annealing

**Figure 6:**
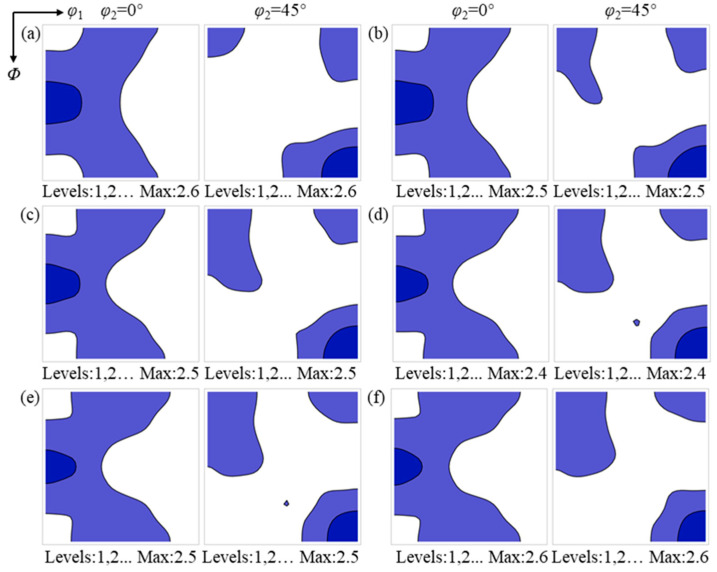
The evolution of texture in the sample surface region during recrystallization: (**a**) 30 s; (**b**) 60 s; (**c**) 95 s; (**d**) 135 s; (**e**) 195 s; and (**f**) 465 s. The color intensity indicates the relative texture strength.

**Figure 7:**
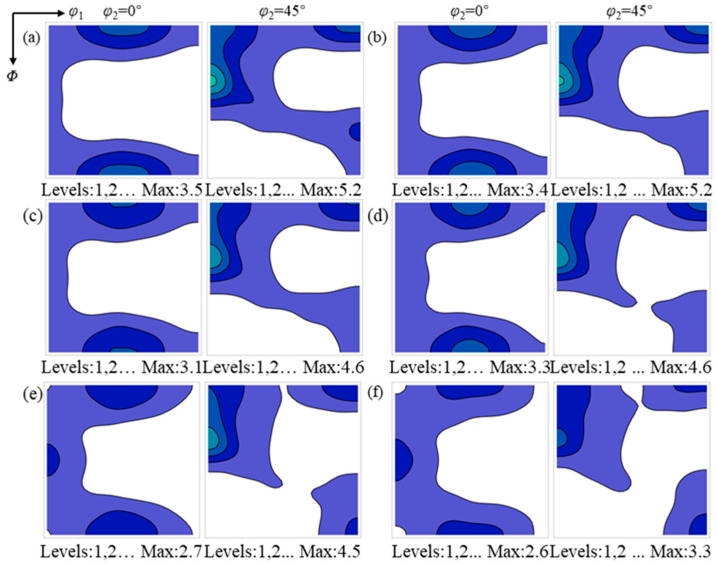
The evolution of texture in the sample center region during recrystallization: (**a**) 30 s; (**b**) 60 s; (**c**) 95 s; (**d**) 135 s; (**e**) 195 s; and (**f**) 465 s. The color intensity indicates the relative texture strength.

Figure 6 and Figure 7 present the ODF sections at φ_2_ = 0° and 45° for the surface and center regions during annealing, respectively. In the surface region, the Goss texture intensity shows no obvious change, while the texture components in the vicinity of the α and α* orientations gradually strengthen, and the {001}<110>~{112}<111> texture weakens. Upon completion of annealing, the surface region is characterized by the coexistence of the Goss texture and relatively uniform α and α* textures. In contrast, in the center region, the α and γ textures gradually weaken with increasing annealing time, whereas the Goss texture progressively strengthens.

The recrystallization nucleation and growth of different texture components occur within deformed matrices of different orientations. By analyzing the temporal evolution of the area fraction, number of grains, and average grain size of recrystallized grains with different texture components, the respective contributions of oriented nucleation and oriented growth to the recrystallization texture can be elucidated.

In this study, the major texture components at the stage when recrystallization of the hot-rolled sheet is essentially completed are analyzed, including {112}<110>, {114}<481>, {001}<210>, and the Goss-oriented recrystallized grains.

**Figure 8:**
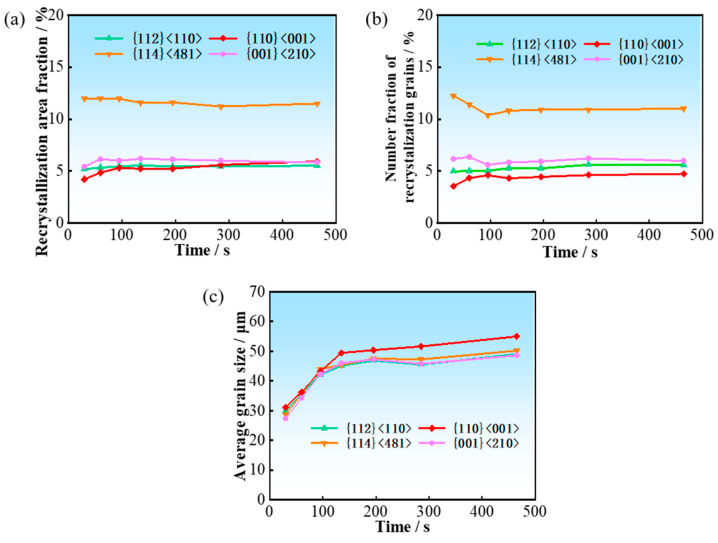
The evolution of area fraction (**a**), grain number (**b**), and average grain size (**c**) for different recrystallization texture components in the surface region.

Figure 8 shows the variation in recrystallization fraction and grain number fraction with respect to all recrystallized grains, as well as average grain size for the four recrystallized texture components during normalization annealing in the surface region. The recrystallization fraction and grain number fraction remains nearly constant, suggesting the dominant role of the oriented nucleation mechanism. The {114}<481> grains have a pronounced numerical advantage owing to their high nucleation rate. Furthermore, various texture components exhibit a similar grain size variation with annealing time. This indicates that there is not an obvious difference in grain growth rate among texture components. Therefore, the area fraction of each texture component is determined by the number of recrystallized grains.

**Figure 9:**
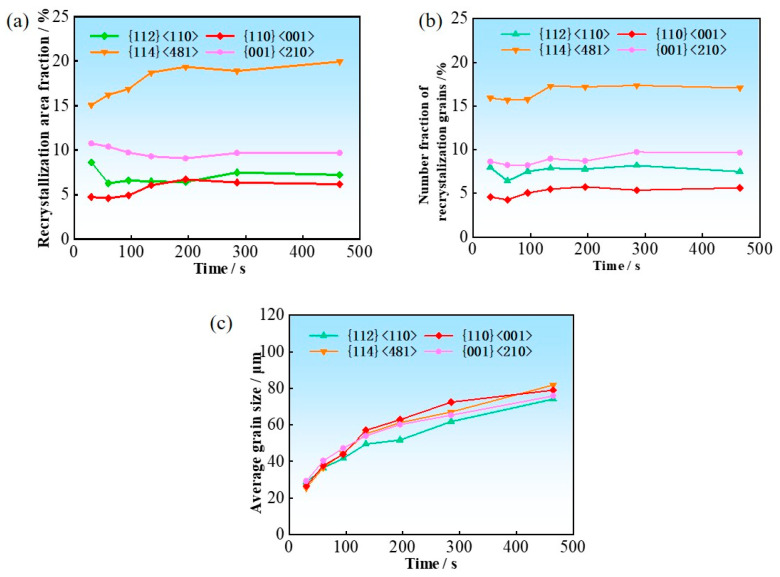
The evolution of area fraction (**a**), grain number (**b**), and average grain size (**c**) for different recrystallization texture components in the center region.

As shown in Figure 9, the evolution of area fraction, grain number and grain size for various texture components in the center region is similar to that in the surface region. No significant differences are observed in the growth rates of grains belonging to different texture components, and the area fractions of the texture components are essentially consistent with the grain number fractions. These results indicate that the recrystallization texture in the center region is generally dominated by the oriented nucleation mechanism.

## 4. Discussion

In this study, the surface and center regions of the hot-rolled sheet exhibit distinct textures and display different recrystallization texture evolution behaviors. The following discussion focuses on elucidating the nucleation and growth behaviors of recrystallized grains corresponding to the major texture components.

### 4.1. The Deformed Matrix Consumed at the Early Stage of Recrystallization

Recrystallized grains with different orientations tend to nucleate and grow within deformed matrices of different orientations, rendering the recrystallization texture sensitive to the initial deformation texture. By analyzing the deformed matrices consumed by recrystallized grains of different orientations during recrystallization, the nucleation and growth behaviors associated with different deformation textures can be elucidated.

**Figure 10:**
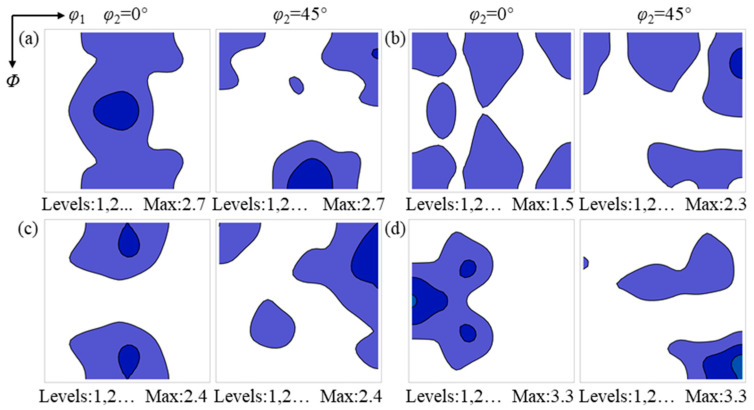
Texture of the deformed matrix consumed by recrystallized grains with different orientations in the surface region after annealing for 60 s: (**a**) {112}<110>, (**b**) {114}<481>, (**c**) {001}<210>, and (**d**) {110}<001>. The color intensity indicates the relative texture strength.

After annealing for 60 s, a considerable number of recrystallized grains have formed in the hot-rolled sheet, while the recrystallized grains have not yet undergone sufficient growth. At this stage, the texture of the deformed matrix consumed by recrystallized grains can, to some extent, reflect the nucleation and early growth environment of recrystallized grains [29]. The texture characteristics of the deformed matrices consumed by the {112}<110>, {114}<481>, {001}<210>, and Goss-oriented recrystallized grains in the surface region are shown in Figure 10.

The {112}<110> recrystallized grains consume deformed matrices with orientations in the vicinity of {001}<110>~{112}<111> and {110}<112>. Both the {114}<481> and {001}<210> recrystallized grains consume deformed matrices with orientations near {001}<110>~{112}<111> and the Goss orientation, whereas the deformed matrix consumed by Goss-oriented recrystallized grains is predominantly located near the Goss orientation.

**Figure 11:**
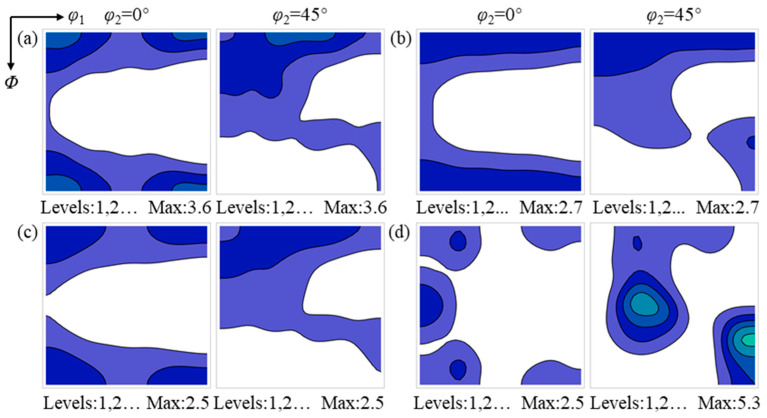
Texture of the deformed matrix consumed by recrystallized grains with different orientations in the center region after annealing for 60 s: (**a**) {112}<110>, (**b**) {114}<481>, (**c**) {001}<210>, and (**d**) {110}<001>. The color intensity indicates the relative texture strength.

The deformation textures consumed by the {112}<110>, {114}<481>, {001}<210> and Goss recrystallized grains in the center region after annealing for 60 s are shown in Figure 11. The {112}<110>, {114}<481> and {001}<210> recrystallized grains mainly consume deformed matrices characterized by relatively uniform α and λ textures, whereas the Goss recrystallized grains consume deformed matrices with orientations in the vicinity of {111}<112>. The deformed matrices consumed by the {112}<110>, {114}<481> and {001}<210> recrystallized grains exhibit similar texture characteristics, indicating that their nucleation occurs in comparable texture environments, which are distinctly different from the texture environment associated with the nucleation of Goss recrystallized grains.

In the surface region, the {112}<110>, {114}<481> and {001}<210> recrystallized grains scarcely consume deformed matrices with orientations close to their own, whereas Goss recrystallized grains consume Goss-deformed matrices during nucleation and early growth. This indicates that Goss recrystallized grains may possess nucleation mechanisms different from those of the {112}<110>, {114}<481> and {001}<210> recrystallized grains. In the center region, the deformed matrices consumed during nucleation and early growth for each texture component differ markedly from those in the surface region, indicating that the same texture component may exhibit different nucleation mechanisms under different environments.

### 4.2. Deformed Matrix Consumed During the Later Stage of Recrystallization

**Figure 12:**
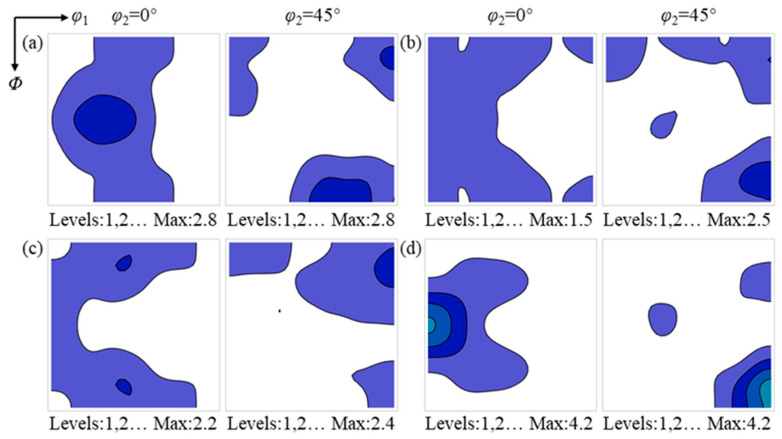
Texture of the deformed matrix consumed by recrystallized grains with different orientations in the surface region after annealing for 465 s: (**a**) {112}<110>, (**b**) {114}<481>, (**c**) {001}<210>, and (**d**) {110}<001>. The color intensity indicates the relative texture strength.

After annealing for 465 s, recrystallization in the hot-rolled sheet is essentially completed. At this stage, the deformed matrices consumed by recrystallized grains of different texture components represent the competitive outcome of recrystallized grain nucleation and growth. By comparing the deformation textures consumed at annealing times of 465 s and 60 s, the competitive relationships among different texture components during nucleation and growth can be elucidated. Figure 12 shows the deformation textures consumed by the {112}<110>, {114}<481>, {001}<210> and Goss recrystallized grains in the surface region, which are similar to those observed after annealing for 60 s. This is because the surface region exhibits a high recrystallization nucleation rate, and recrystallized grains come into contact with neighboring recrystallized grains after only a limited extent of growth following nucleation. Subsequently, the driving force for grain growth shifts from deformation-stored energy to grain boundary energy. As recrystallized grains do not undergo sufficient growth under the driving force of deformation-stored energy, potential oriented growth effects do not become apparent. The deformation texture-dependent nucleation and early growth of recrystallized grains therefore determine the recrystallization texture in the surface region.

**Figure 13:**
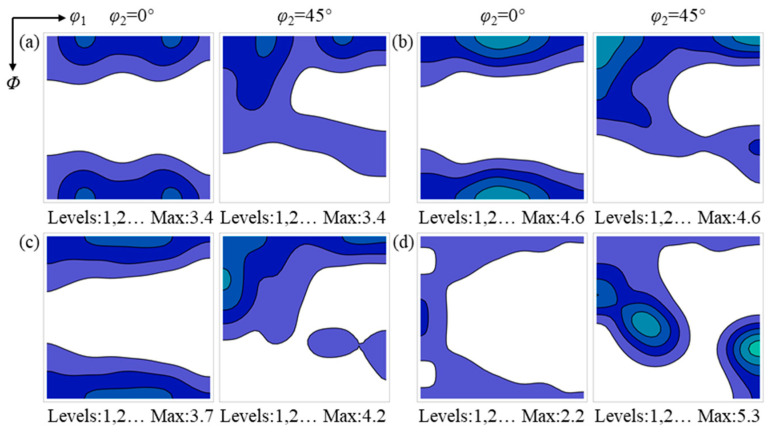
Texture of the deformed matrix consumed by recrystallized grains with different orientations in the center region after annealing for 465 s: (**a**) {112}<110>, (**b**) {114}<481>, (**c**) {001}<210>, and (**d**) {110}<001>. The color intensity indicates the relative texture strength.

Figure 13 presents the deformation textures consumed by the {112}<110>, {114}<481>, {001}<210> and Goss recrystallized grains in the center region. The types of deformation textures consumed by the {112}<110>, {114}<481> and {001}<210> recrystallized grains are similar to those observed after annealing for 60 s; however, the texture intensity peak of the consumed deformed matrices vary with the orientations of the recrystallized grains. Specifically, the deformation texture intensity maxima consumed by the {112}<110>, {114}<481> and {001}<210> recrystallized grains are located near the {001}<210>, {001}<110> and {113}<110>, respectively. The deformation texture intensity peak consumed by Goss recrystallized grains remains near the {111}<112>, which may be attributed to the formation of highly mobile grain boundaries between Goss grains and the {111}<112> [30].

In the center region, the nucleation density of recrystallized grains is low, and grains of different texture components undergo extensive growth under the driving force of stored energy during recrystallization. The {112}<110>, {114}<481> and {001}<210> recrystallized grains consume similar deformed grains during nucleation and early growth, whereas during subsequent growth, they consume different deformed matrices; that is, recrystallized grains with specific orientations tend to consume deformed matrices with corresponding specific orientations. These observations indicate that the oriented growth mechanism may contribute to the evolution of the recrystallization texture in the center region.

### 4.3. Recrystallized Grain Growth Behavior

When recrystallization in the center region of the hot-rolled sheet is completed, recrystallized grains with different orientations tend to consume deformed matrices with different orientations, indicating that oriented growth occurs during recrystallization [31]. Building upon this general observation, the present results further demonstrate that recrystallized grain growth in the center region of the hot-rolled silicon steel exhibits a pronounced orientation dependence, with distinct recrystallized orientations preferentially consuming specific deformed matrix orientations. Such behavior suggests that the crystallographic character of the recrystallization front plays an evident role during grain growth.

In this context, the involvement of specific CSL grain boundaries provides a plausible mechanistic framework for interpreting the observed orientation-dependent consumption behavior. Table 2 gives the grain boundary type between large-sized grains and their main deformed matrix. The high mobility of Σ9 grain boundaries in silicon steel has been well established by direct grain boundary migration measurements [32], which offers a consistent explanation for the preferential consumption of {111}<112> deformed matrices by Goss recrystallized grains observed in this study. Although direct experimental evidence for the enhanced mobility of Σ7 grain boundaries in silicon steel is still lacking, previous studies have shown that near-Σ7 orientation relationships are frequently associated with oriented growth during recrystallization [33,34,35]. Taken together, these observations suggest that Σ7 grain boundaries may contribute to the selective consumption of {001}<210> and {113}<110> deformed matrices by {112}<110> and {001}<210> recrystallized grains, respectively. Sanjari et al. [36,37] pointed out that near-Σ13b grain boundaries may be responsible for the formation of large-sized {113}<121> recrystallized grains originating from deformed {001}<130> grains. Based on the observation, it can be inferred that Σ13b grain boundaries possess a mobility advantage and facilitate the consumption of the {001}<110> deformed matrix by {114}<481> recrystallized grains.

The orientation-dependent consumption behavior revealed in this work highlights the possible role of grain boundary crystallography in recrystallized grain growth in hot-rolled silicon steel and provides insight into the origin of recrystallization textures in the center region of the hot-rolled sheet. However, since the stored energy is also an important factor for grain boundary motivation rate, a further measurement is needed to more explicitly elucidate the relative contribution from boundary mobility and stored energy in the orientation-dependent consumption of deformed matrices during recrystallization.

## 5. Conclusions

The microstructural and textural evolution during the recrystallization of hot-rolled silicon steel sheets was systematically investigated. The main conclusions are summarized as follows:

(1) The formation of the recrystallization texture in the hot-rolled sheet is governed primarily by the oriented nucleation mechanism. During recrystallization, the Goss texture intensity in the surface region remains nearly unchanged, whereas the α and α* textures are strengthened. In the center region, the α texture is weakened, the α* texture shows little variation, and the Goss texture becomes enhanced.
(2) Recrystallized grains with different orientations nucleate by consuming deformed matrices with different orientations. In the surface region, {112}<110> recrystallized grains nucleate by consuming deformed matrices near {114}<221> and {110}<112>, while {114}<481> and {001}<210> recrystallized grains consume deformed matrices near {114}<221> and Goss orientations. Goss-oriented recrystallized grains mainly consume Goss-oriented deformed matrices. In the center region, {112}<110>, {114}<481>, and {001}<210> recrystallized grains nucleate by consuming α- and λ-type deformed matrices, whereas Goss recrystallized grains consume deformed matrices with orientations near {111}<112>.
(3) In the center region, the deformed matrices consumed by recrystallized grains during the late stage of recrystallization depend strongly on the orientation of the recrystallized grains. The {112}<110>, {114}<481>, {001}<210>, and Goss recrystallized grains preferentially consume deformed matrices with orientations near {001}<210>, {001}<110>, {113}<110>, and {111}<112>, respectively.
(4) The selective growth of recrystallized grains in the center region may be related to the presence of special grain boundaries. The involvement of Σ9 grain boundaries, which exhibit relatively high mobility in silicon steel, offers a reasonable interpretation of the observed preferential growth behavior. Moreover, Σ7 grain boundaries may also play a role in orientation-dependent recrystallized grain growth during annealing.

## Figures and Tables

**Table 1:** Chemical composition of the silicon steel used in this study (wt.%).

C	Si	Mn	P	S	Fe
0.003	3.02	0.27	0.009	0.0007	Bal.

**Table 2:** Grain boundary type between large-sized grains and the main deformed matrix.

Recrystallized Grain	Deformed Matrix	Grain Boundary	Misorientation Angle (°)
{112}<110>	{001}<210>	Σ7	7.4
{114}<481>	{001}<110>	Σ13b	5.6
{001}<210>	{113}<110>	Σ7	8.1
Goss	{111}<112>	Σ9	3.9

## Data Availability

The original contributions presented in the study are included in the article, and further inquiries can be directed to the corresponding authors.
